# Supplementary material for: Identification of photoperiod-induced specific miRNAs in the adrenal glands of Sunite sheep (Ovis aries)
Source: Front Vet Sci. 2022 Jul 22;9:888207. doi: 10.3389/fvets.2022.888207 (PMC9354845; doi:10.3389/fvets.2022.888207)
Supplement: Supplementary file 1 [file Data_Sheet_1.ZIP › Supplementary file/miRDeep2 file.zip!/miRDeep2 file/Know_Structure/SPLP42A3/oar-mir-376c.pdf]

miRBase precursor : oar-mir-376c
Total read count : 593
oar-miR-376c-5p read count : 342
oar-miR-376c-3p read count : 249
remaining reads : 2

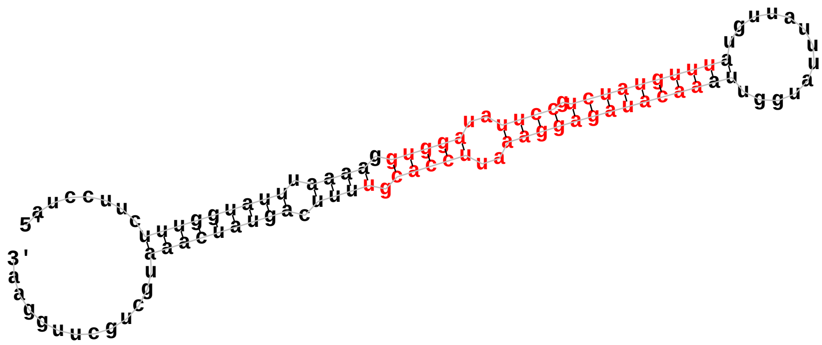

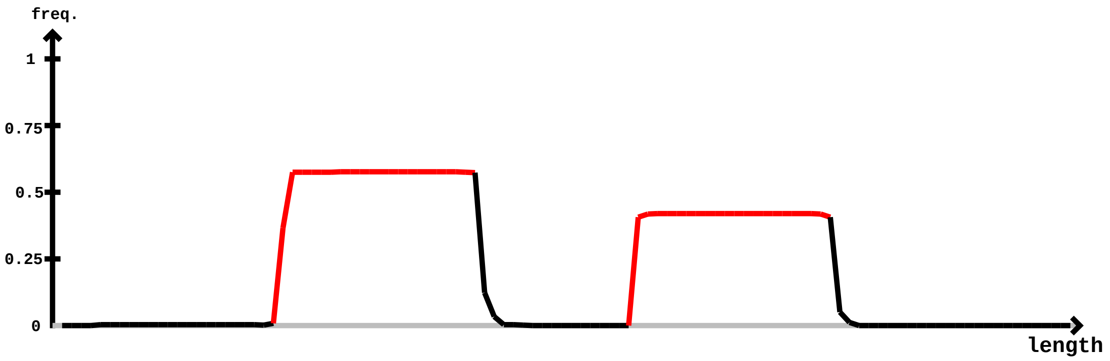


```
                                                                       oar-miR-376c-3p
                                  oar-miR-376c-5p
5'- auccuucuuugguauuuaaaagguggauauuccgucuauguuuauguuauuuaugguuaaacauagaggaaauuccacguuuucaguaucaaaugcugcuuggaa -3'  exp
    .......(((((((((.((((.(((((..((((.((((((((((.............))))))))))))))..))))).)))).)))))))))............   reads  mm  sample
    ...cuucuuugguauuuaaa.....................................................................................   1      0   seq
    ...cuucuuugguauuuaaaag...................................................................................   1      0   seq
    .....................gguggauauuccgucuauguuu..............................................................   4      0   seq
    ......................guggauauuccgucuauguu...............................................................   1      0   seq
    ......................guggauauuccgucuauguuu..............................................................   163    0   seq
    ......................guggauauuccgucuauguuua.............................................................   43     0   seq
    ......................guggauauuccgucuauguuuau............................................................   6      0   seq
    .......................uggauauuccgucuaugu................................................................   1      0   seq
    .......................uggauauuccgucuauguuu..............................................................   100    0   seq
    .......................uggauauuccgucuauguuua.............................................................   10     0   seq
    .......................uggauauuccgucuauguuuau............................................................   12     0   seq
    .......................uggauauuccgucuauguuuaugu..........................................................   1      0   seq
    ............................auuccgucuauguuuauguu.........................................................   1      0   seq
    ...........................................................aacauagaggaaauuccac...........................   1      0   seq
    ...........................................................aacauagaggaaauuccacg..........................   7      0   seq
    ...........................................................aacauagaggaaauuccacgu.........................   211    0   seq
    ...........................................................aacauagaggaaauuccacguu........................   21     0   seq
    ...........................................................aacauagaggaaauuccacguuu.......................   1      0   seq
    ............................................................acauagaggaaauuccacguu........................   2      0   seq
    ............................................................acauagaggaaauuccacguuu.......................   5      0   seq
    .............................................................cauagaggaaauuccacguuu.......................   1      0   seq
```
